# Densification and Phase Transformation in Multi-Layered Graded Si_3_N_4_–TiN Components Produced by Field-Assisted Sintering

**DOI:** 10.3390/ma12182900

**Published:** 2019-09-08

**Authors:** Dong-Tao Lin, Li-Juan Yuan, Peng-Jie Zhang, Fei Zuo, Kevin Plucknett, Salvatore Grasso, Hong-Jian Wang, Hua-Tay Lin

**Affiliations:** 1 School of Electromechanical Engineering, Guangdong University of Technology, Guangzhou 510006, China; 2111701217@mail2.gdut.edu.cn (D.-T.L.); EchoYLJ@163.com (L.-J.Y.); zpj19960116@outlook.com (P.-J.Z.); kevin.plucknett@dal.ca (K.P.); wanghongjian1105@163.com (H.-J.W.); 2 Department of Mechanical Engineering, Dalhousie University, Halifax, NS B3H4R2, Canada; 3 Key Laboratory of Advanced Technologies of Materials, Southwest Jiaotong University, Chengdu 610031, China; s.grasso@swjtu.edu.cn

**Keywords:** field-assisted sintering technique (FAST), Si_3_N_4_-based composites, thermodynamics, functionally gradient material (FGM), current waveform, particle size

## Abstract

The structural and/or functional design of multiphase ceramics, along with their processing, are timely research topics in the area of field-assisted sintering techniques, such as spark plasma sintering, especially for systems containing both electrically insulating and conductive phases. In the present study, spark plasma sintering of Si_3_N_4_–TiN composites was investigated by changing the TiN particle size and electrical current waveform. Their combined effects on both the densification behavior and α-to-β phase conversion of the Si_3_N_4_ matrix was studied and compared by means of a thermodynamic approach and dilatometric measurements. Through the control of TiN phase characteristics and heating mode, double-layered Si_3_N_4_-based components were also prepared using a one-step spark plasma sintering process, which was compared with conventional hot-pressing. It was shown that the size of the conductive TiN phase has a significant influence on the particle rearrangement, with the formation of a liquid phase, and the solution–diffusion–precipitation process, through the field-induced local heating and electrowetting mechanisms. Moreover, the contribution of current pulsing to the densification and α-to-β conversion of the layered Si_3_N_4_-based components was mostly dependent upon the particle size distribution and content of the TiN phase, indicating that the electric-field effect is dependent upon current path.

## 1. Introduction

Based on conventional pressure-assisted sintering techniques, spark plasma sintering (SPS) has been developed with the purpose of fully densifying materials, such as nitride and carbide ceramics, that are predominantly covalently bonded and have low atomic self-diffusion [1,2,3]. In addition, by optimizing sintered densities, a predictable and effective control of the phase composition and microstructure of the processed materials would also be significant benefits expected when using this economical sintering method [4,5,6]. Unlike oxide ceramics, those based on nitrides and carbides are routinely consolidated using liquid phase sintering (LPS). As the liquid phase forms during heating of a powder mixture, a series of concomitant effects will contribute to the densification and microstructure development. These include, liquid flow, solution–diffusion–reprecipitation, and solid-state sintering. The mechanism(s) responsible for the field-induced phenomena in LPS are still debated in the ceramic community. Consequently, a challenging question that draws considerable attention from researchers is whether there is a field-induced effect when applying SPS, beyond that achieved from the rapid heating [7,8].

Under the action of external electric or electromagnetic fields, the heterogeneity of the different phases should be considered as a significant factor, potentially affecting the field-induced phenomena by varying the degree of coupling, consequently changing the distributions of the electric field and temperature within the constituents of the composite [9,10]. In this sense, it is likely that the presence of an electrically conductive second phase, within an electrically insulating matrix, could help to clarify the field-assisted constitutive mechanism(s) controlling LPS of multiphase ceramic composites. In a recent investigation, Zuo et al. reported that the applied electric current waveform could significantly influence the densification process, α- to β-Si_3_N_4_ conversion, and microstructure evolution of Si_3_N_4_ matrix material through the addition of a conductive secondary phase when using SPS processing [11]. However, the potential differences in mechanism controlling the field-induced phenomena still remain open for significant and comprehensive investigations. From another perspective, although SPS has been considered as an effective means for developing new Si_3_N_4_-based functionally gradient materials (FGMs), the majority of existing studies have been restricted to using the thermal effect of the graphite mold or the fast heating rates to achieve the graded Si_3_N_4_ structure [5,12,13,14]. In order to extend the technological possibilities to spatially controlling the phase composition and microstructure within Si_3_N_4_-based materials, by varying the current waveform and conductive phase parameters in a one-step SPS process, there is a need to increase understanding of the phenomena that take place during the sintering of graded Si_3_N_4_-based components, as well as the underlying mechanism.

The apparent activation energy (*E_a_*) for either the densification or phase conversion processes are critical thermodynamic parameters. They can provide an empirical insight into the different mechanism(s) that may be involved during sintering of Si_3_N_4_-based ceramics. Although there have been a few investigations on the evaluation of *E_a_* for α- to β-Si_3_N_4_ conversion during SPS of Si_3_N_4_ ceramics [15,16], to the best of the authors’ knowledge, no detailed work has yet been reported concerning the thermodynamic approach undertaken by combined modification of the material and process factors. Specifically, this might include incorporating a conductive phase, varying its morphology or size, and altering the electric current waveform, which seems most likely to interfere with the “electric-field effect” in the SPS process. Accordingly, the present work aims to systematically investigate the influence of particle size distribution of the TiN conductive phase, along with the electric current waveform, on the apparent values of *E_a_* for the α- to β-Si_3_N_4_ conversion during SPS processing of Si_3_N_4_–TiN composites. It should also be noted that multi-layered ceramics are important materials in a wide-range of applications. Therefore based on this foundation, the exploratory application and potential value of the SPS technique for design and fabrication of double-layered Si_3_N_4_-based ceramics will be discussed in the present work. In view of this fundamental and applied approach, field-assisted constitutive mechanism(s) controlling the densification and phase transformation of Si_3_N_4_-based ceramics have been proposed when utilizing the SPS process. This approach offers great opportunities to design multiphase functionally-graded components by taking advantage of the specific features of field-assisted sintering techniques.

## 2. Experimental Procedures

**Figure 1:**
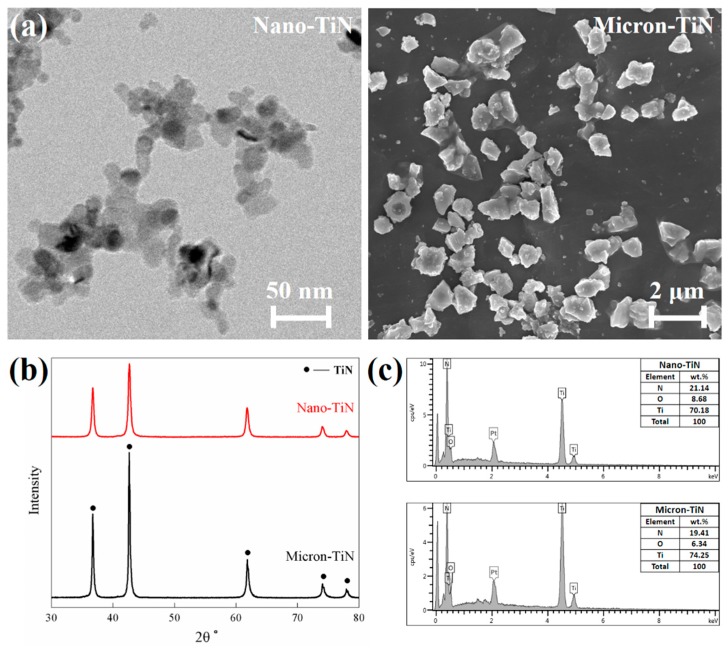
Basic characteristics of the as-received TiN particles: (**a**) electronic micrographs [11]; (**b**) X-ray diffraction (XRD) patterns; and (**c**) energy dispersive X-ray spectrometer (EDS) analysis.

The silicon nitride matrix system was prepared by mixing and milling 90 wt % Si_3_N_4_ (SN-E10, α-Si_3_N_4_ >95%, specific surface area: 11 m^2^/g, UBE, Tokyo, Japan) powders with 6 wt % Y_2_O_3_ (purity: 99.9%, mean particle size: ~150 nm, Fandechen, Beijing, China) and 4 wt % Al_2_O_3_ (TM-DAR, mean particle size: 100 nm, Taimei, Tokyo, Japan) in ethanol, using a planetary ball mill with Si_3_N_4_ media, for a duration of 6 h. TiN powders (Kaier Nano, Hefei, China), with different particle sizes (30 nm and 1 μm), were used as the conductive secondary phase (Figure 1). The Si_3_N_4_–TiN mixtures were prepared by adding 10–30 vol % (20 vol % for the densification and thermodynamic studies; while 10 and 30 vol % for the fabrication of multi-layered graded ceramics) of TiN powders into the Si_3_N_4_–Y_2_O_3_–Al_2_O_3_ matrix slurry, and then ball-milling for 24 h. In order to avoid any remarkable particle size reduction, and also to maintain a uniform distribution of the TiN phase within the Si_3_N_4_ matrix, relatively large Si_3_N_4_ balls with 5–8 mm diameter and a low milling speed of 90–110 rpm were employed. The milling was carried out at a ball to powder weight ratio of 3:1. The slurry mixtures were subsequently dried in a rotary evaporator and then passed through a 120-mesh sieve to eliminate any hard aggregates.

Field-assisted sintering experiments were conducted with a SPS apparatus (HPD10, FCT, Frankenblick, Germany). To allow direct comparison of the shrinkage curves for each sample, the Si_3_N_4_–TiN powder blend mass added into the graphite SPS die, with an inner diameter of 20 mm, was kept constant for all tests. Graphite foil was used to separate the mold/punch surfaces from the powder mixtures to ensure a good electrical contact and prevent any reaction between the samples and the graphite SPS tooling. The temperature during SPS was measured from the top by a monochromatic pyrometer. Isothermal dwell holds, for various times (from 0 to 1200 s, referring to the data plotted in the section of Scale-Effect of Electric Field-Induced α–β Conversion), were carried out separately at 1650, 1700, 1750, and 1800 °C. In all cases, the heating rate was 50 °C/min, under an applied load of 30 MPa, in a N_2_ atmosphere. SPS conditions of continuous direct current (DC) power or pulsed DC power (10:5 milliseconds on:off) were used on the powder mixtures. The dilatometric data during SPS were continuously monitored by an extensometer integrated with the loading rod head. The double-layered composites were prepared through both conventional hot-pressing (HP, VHP200, Weitai, Shenyang, China) and SPS at 1700 °C for 5 min in N_2_ atmosphere under 30 MPa, at heating rates of 20 and 50 °C/min, respectively. 

Scanning electron microscopy (SEM, SU8220, Hitachi, Tokyo, Japan) was used for the observation of the microstructures of polished and plasma-etched samples. An energy dispersive X-ray spectrometer (EDS) was used with the SEM for analysis of chemical composition. X-ray diffraction (D8 ADVANCE, Bruker, Karlsruhe, Germany) was applied for the determination and quantization of the α- and β-Si_3_N_4_ phases in the sintered samples by means of the Rietveld method [17]. At least two different specimens for each composition, and two X-ray diffraction (XRD) tests for each specimen were carried out for consistency.

## 3. Results and Discussion

### 3.1. Scale-Effect of Electric Field-Induced Densification

**Figure 2:**
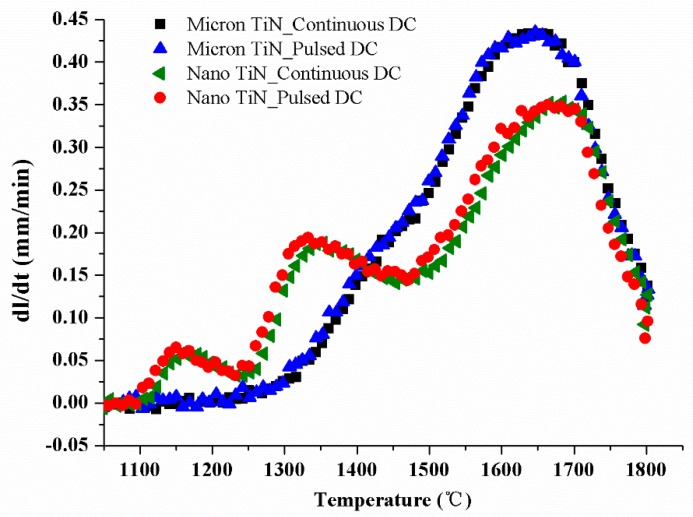
Typical examples of the shrinkage rates curves obtained during current-assisted sintering of Si_3_N_4_-20 vol % TiN composites (samples held at 1800 °C for 30 s), when using different TiN particle sizes and direct current (DC) waveforms.

Typical examples of the dilatometry data are shown in Figure 2, highlighting the shrinkage rates of the various Si_3_N_4_-20 vol % TiN composites sintered at 1800 °C with a 30 s dwell time. For composites containing the micron-sized TiN as the conductive second phase, the curves under both applied current waveforms present evidence of two convoluted peaks, which are associated with the two important stages of LPS, namely the α-Si_3_N_4_ particle rearrangement by liquid flow (present at about 1440 °C as a shoulder on the primary peak) and the material dissolution into and precipitation out from a liquid phase (the primary peak at about 1650 °C) [18]. As the TiN particle size is decreased, however, the shrinkage initiation temperatures and curve shapes are clearly distinguishable from the case with larger TiN. It is notable that, for each individual TiN particle size, the shrinkage rate curves follow the same path with no significant dependency on the electric current waveform.

Dimensional change of the LPS-processed composites was initially induced by liquid formation, as well as its flow due to an instantaneous capillary action within the pores between particles. Liquid formation should occur at a nominally constant temperature when the LPS additives have the same chemical composition. By means of XRD and EDS analysis on the initial TiN powders (Figure 1), both the nano- and micron-TiN powders showed single phase character, and their oxygen impurities were at an essentially identical level. The conventional HP of the composites containing 20 vol % of nano-TiN and micron-TiN were separately carried out at heating rate of 20 °C/min as well (not shown here), but indicates the shrinkage of both started at 1350–1400 °C (apparent temperature) for the Y_2_O_3_ (6 wt %):Al_2_O_3_ (4 wt %) additive system. Thus, there appear to be different shrinkage start temperatures when comparing the SPS and HP processes. The different SPS-processed specimens might all reflect their differing coupling extents between the imposed current (i.e., external electric field) and composite composition/microstructure, which would directly determine the local temperature of the liquid phase at the grain boundaries and could then bring about the so-called “overheating effect” [19]. Compared with Si_3_N_4_ (with a resistivity of ~10^12^ Ω·m), the electrical resistivity of TiN is much lower (~10^–7^ Ω·m). Therefore, the Si_3_N_4_–TiN composites would couple with the current and be locally heated by mechanisms including plasma (discharge) generation and/or the Joule effect. When the TiN particle size reduces from the micron-scale to nano-scale, its distribution extent at grain junctions and grain boundaries within the Si_3_N_4_ matrix would increase, causing more areas of localized heating. Consequently, the reduction of TiN particle size contributed to an earlier shrinkage taking place, at a temperature lowered by ~200 °C. In addition, the conductive TiN particles located in the non-conductive Si_3_N_4_ matrix would also exist as an effective, contiguous current path under the action of the applied electric field, and thus significantly improve the effectiveness and uniformity of the electromechanical forces acting on wetting the liquid on the Si_3_N_4_ particles [11,20]. The distribution of liquid at the neck areas between particles, as well as particle rearrangement, could therefore be significantly facilitated by reduction of the TiN particle size from the micron- to nano-scale. Nevertheless, it should be noted that both “overheating” and “electrowetting” are locally enhanced effects. Their final contributions to the liquid-induced particle rearrangement would be also governed by the formation, viscosity, distribution, and homogeneity of the oxynitride liquid phase. For this reason, after the appearance of the first particle rearrangement peak at ~1160 °C, a second peak at ~1350 °C is apparent on the shrinkage rate curves of the Si_3_N_4_ samples with nano-TiN. The effect of TiN particle size on the reduction of the particle rearrangement peak temperature was consequently quite evident.

### 3.2. Scale-Effect of Electric Field-Induced α–β Conversion

**Figure 3:**
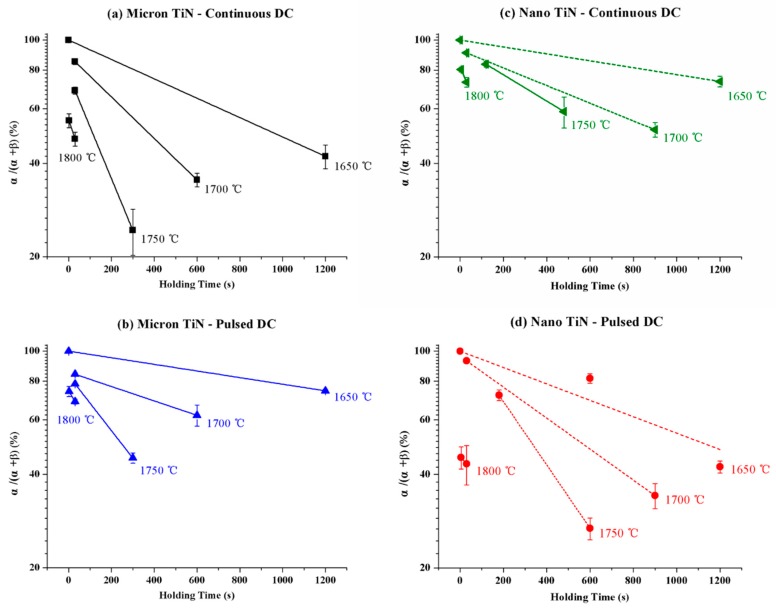
Time dependence of the degree of Si_3_N_4_ α-to-β phase conversion fraction for Si_3_N_4_-20 vol % TiN ceramic composites with (**a**,**b**) micron- or (**c**,**d**) nano-TiN particles, respectively sintered under (**a**,**c**) continuous current mode or (**b**,**d**) pulsed mode; each data point represents the average value, with associated standard deviation error, for current-assisted sintering samples held at a specific temperature for a set dwell time.

**Figure 4:**
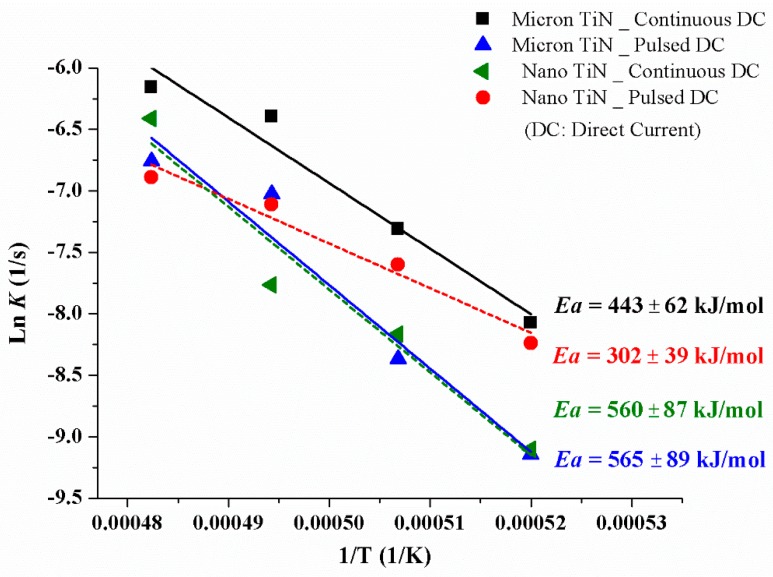
Arrhenius plot of values of the rate constant, *K*, for the α-to-β phase conversion of the Si_3_N_4_ matrix during isothermal holds, for Si_3_N_4_-20 vol % TiN samples sintered in the temperature range of 1650–1800 °C.

In order to further reveal the effects of the current waveform and conductive phase in electric current-assisted LPS of the Si_3_N_4_-20 vol % TiN composites, the extent of α- to β-Si_3_N_4_ conversion in the isothermal sintering process was examined. The time dependence of the phase transformation degree is presented in Figure 3 for various SPS temperatures. The data suggest that the conversion follows first-order kinetics, in accordance with previous observations reported in the literature [15,21,22]. Thus, the α–β phase conversion rate of the Si_3_N_4_ matrix can be expressed using the equation:

(1) $$\frac{d\alpha }{dt}=-K\alpha$$

where α is the α-Si_3_N_4_ fraction in the composite, and *K* is the rate constant dependent only on the sintering temperature, which can itself be defined by the following equation:

(2) $$K=K_{0}\exp\left(\frac{E_{a}}{RT}\right)$$

where *Ea* is the apparent activation energy, *R* is the gas constant, and *T* is the absolute temperature. Figure 4 demonstrates the temperature dependence of the phase transformation in the form of an Arrhenius plot, constructed using the rate constant *K* values derived from the data in Figure 3. From this information, the *Ea* values are obtained from the slope of the regression line for each sample. It can be seen that a change in the current waveform or the particle size of the conductive phase produces significant changes in the activation energy for the α–β phase conversion of the Si_3_N_4_ matrix phase. According to the model proposed by Bowen et al. [21], it is suggested that both densification and phase transformation of Si_3_N_4_ can take place simultaneously in conventional hot processing, as atoms pass through the dissolution–diffusion–precipitation cycle. Thus, the activation energies for both the densification (during heating) and conversion processes (during dwelling) are quite similar in their study [21]. In the present case, however, the external electric field may lead to different effects on the particle rearrangement and dissolution–precipitation processes. In this sense, the apparent activation energies evaluated in Figure 4, and the shrinkage rate curves plotted in Figure 2, could be analyzed relatively independently. 

**Figure 5:**
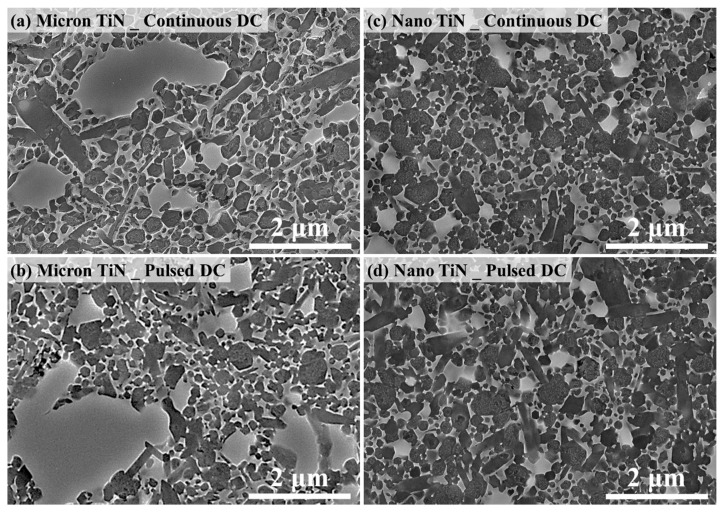
Microstructural images of Si_3_N_4_-20 vol % TiN samples with (**a**,**b**) micron- or (**c**,**d**) nano-TiN particles, respectively sintered under (**a**,**c**) continuous current mode or (**b**,**d**) pulsed mode at 1800 °C for 30 s.

**Figure 6:**
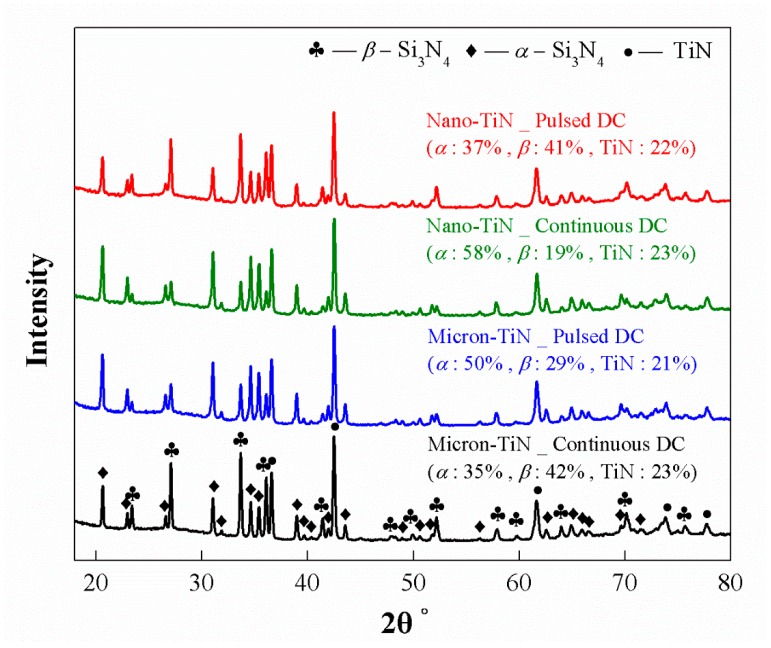
XRD patterns with qualitative and quantitative analysis of Si_3_N_4_-20 vol % TiN samples with micron- or nano-TiN particles, respectively sintered under continuous current mode and pulsed mode at 1800 °C for 30 s.

Whether for the samples with micron-TiN sintered under continuous current conditions, or for those with nano-TiN sintered under pulsed current mode, the apparent *E_a_* for α–β phase conversion are very similar, both around 560 kJ/mol. This value coincides with that evaluated for hot-pressed Si_3_N_4_ made with 5 wt % Y_2_O_3_ and 3 wt % Al_2_O_3_ sintering additives [15]. But once the SPS current pulsing mode changed, the apparent Ea values varied significantly in the present work. The apparent *E_a_* for the samples with micron-TiN, sintered under continuous current mode, is ~120 kJ/mol lower than that under pulsed mode, whereas the samples containing nano-TiN phase inverted this trend. To confirm the thermodynamic evaluation, Figure 5 shows examples of SEM micrographs of polished and plasma etched cross-sections of the SPS processed materials. The images demonstrate the differences in microstructure evolution for specimens isothermally sintered at 1800 °C for 30 s with both different TiN particle sizes and electric current waveform conditions, while Figure 6 demonstrates the XRD patterns with qualitative and quantitative analysis on the corresponding samples. Figure 5 and Figure 6 clearly indicate the effects of the same current pulsing mode on the α- to β-Si_3_N_4_ conversion when different TiN particle sizes are used. Thus, it is confirmed that the electric-field effect should be particle size-dependent in the presence of a conductive second phase. 

In the LPS process, the amount, chemical composition, and viscosity of the liquid phase each have a distinct influence on the initiation and completion of the α- to β-Si_3_N_4_ conversion, certainly including the activation energy [23,24,25]. For instance, the *E_a_* for diffusion in a Si-RE-Mg oxynitride viscous phase boundary region was reported as being 690 kJ/mol and 450 kJ/mol below and above 1550 °C, respectively, since the viscosity decreases dramatically with temperature [21,25]. As a debatable issue in SPS processing, the Joule effect and local generation of plasma are usually considered to play major roles on the SPS heating mechanisms [7,8]. However, one or some combination of the possible mechanisms should become more dominant under certain conditions [26]. Under pulsed current mode, the instantaneous voltage at the bonding interface is expected to be higher, relative to the value under the continuous current sintering mode [11,27,28]. In particular, for the small conductive particle size, a high electric field strength may be achieved at the nano-scale contact areas. Thus, for the nano-TiN, owing to its fine particle size (about 30 nm) and wide distribution within the Si_3_N_4_ matrix (about 180 nm), it could have increased the possibility to generate local heating between the powder particles. Instead, as the particle size of the electrically insulating matrix phase becomes somewhat smaller than that of the conductive phase (i.e., micron-scale TiN (about 1 μm) in the Si_3_N_4_ matrix), the local Joule heating effect induced by the continuous DC passing through the composite would provide major contributions to govern both the densification and α–β phase conversion processes. However, while further fundamental research is necessary to clarify this assumption on the dominant mechanism(s) controlling the electric-current-assisted sintering process, the present dilatometric analysis and thermodynamic approach give clear insight that the contribution of the current mode on the field-induced densification and phase conversion of the electrically insulating matrix would depend mostly on the particle size and distribution of the electrically conductive phase. The non-uniform local electrical properties within these multiphase ceramics would control the local electric field and temperature distributions, which then govern the various phenomena involved in the SPS process.

### 3.3. Design and Fabrication of Multi-Layered Graded Si_3_N_4_–TiN Ceramics

**Figure 7:**
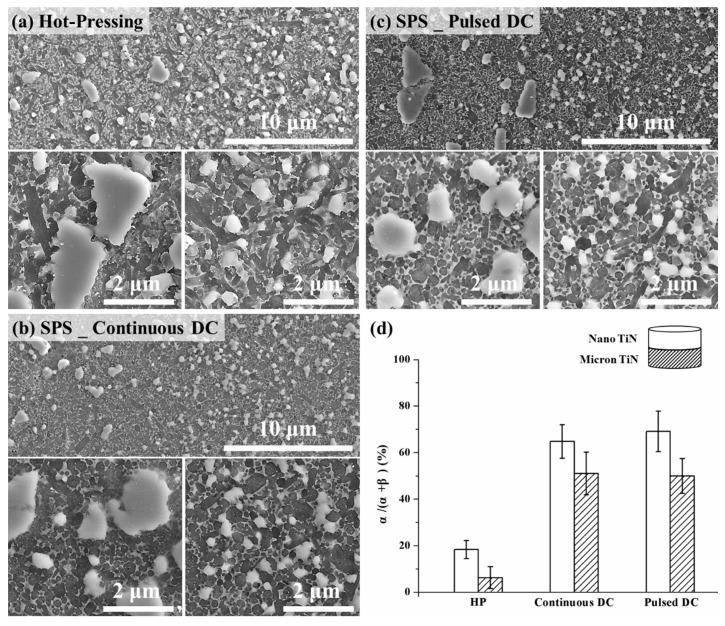
Low (upper side, including the layer interface) and high (underside) magnification cross-section views of the double-layered Si_3_N_4_-10 vol % TiN composites, prepared at 1700 °C for 5 min with nano- and micro-TiN, by using (**a**) hot-pressing, (**b**) spark plasma sintering (SPS) continuous mode, and (**c**) SPS pulsed mode; and (**d**) the α-Si_3_N_4_ fraction within each layer.

**Figure 8:**
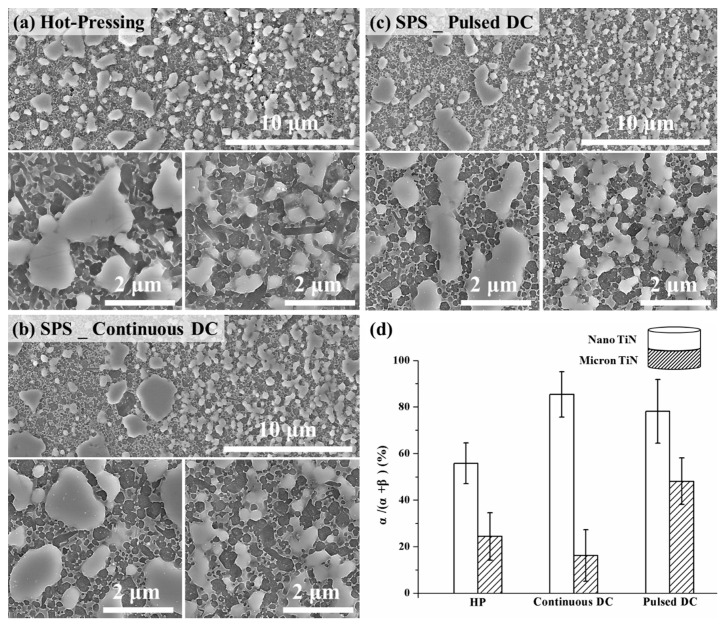
Low (upper side, including the layer interface) and high (underside) magnification cross-section views of the double-layered Si_3_N_4_-30 vol % TiN composites, prepared at 1700 °C for 5 min with nano- and micron-TiN, by using (**a**) hot-pressing, (**b**) SPS continuous mode, and (**c**) SPS pulsed mode; and (**d**) the α-Si_3_N_4_ fraction within each layer.

**Figure 9:**
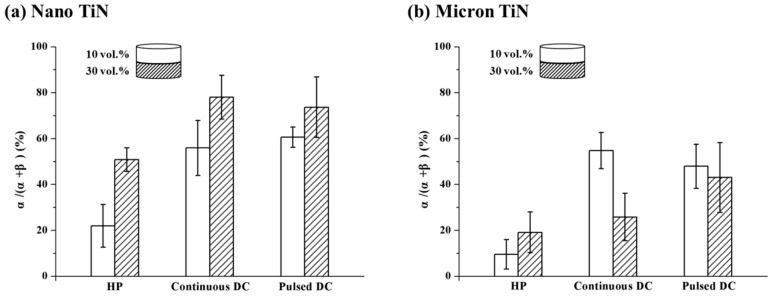
The α-Si_3_N_4_ fraction of each layer within the double-layered Si_3_N_4_–TiN composites with (**a**) nano-sized TiN and (**b**) micron-sized TiN, prepared at 1700 °C for 5 min by using different sintering methods. Each layer contains different TiN contents.

Multi-layered structures are one of the most common forms in the design of one-dimensional FGMs [29,30]. In order to clarify the potential applications of the scale-effect of a conductive second phase on the fabrication of multi-layer, graded Si_3_N_4_-based ceramics through SPS, four groups of double-layered samples were prepared by varying the TiN particle size in the two adjacent layers together with the DC mode. These two layer composite variants were also prepared with either 10 or 30 vol % TiN in the layers, as shown in Figure 7, Figure 8 and Figure 9. It should be noted that the apparent activation energies for α–β phase conversion were evaluated only for Si_3_N_4_ composites prepared with 20 vol % TiN, a composition at which the sintering response could demonstrate an obvious difference with changes in the pulsing mode and TiN particle size [11]. This gives a fundamental viewpoint on the possible mechanism(s) that generate the differing responses. From the perspectives of application and validation, as discussed in the present section, the amount of TiN conductive phase used should be extended both above and below 20 vol %. In doing so, the basic mechanism(s) could be better understood and applied. Based on the SEM micrographs presented in Figure 7 and Figure 8, the use of SPS processing is beneficial to maintain both the α-Si_3_N_4_ phase and a finer grain structure, due to the shorter heating time. In addition, all of the two-layered structures were well bonded and free of defects within each layer, independent of the heating methods applied. However, with changes in the size parameters of the TiN phase, there were significant differences in the α- to β-Si_3_N_4_ conversion degree among the three kinds of sintering modes. 

Based on Figure 7d and Figure 8d, compared with the layers containing micron-TiN second phase, the layers with TiN at the nano-scale showed lower conversion degrees of the α-Si_3_N_4_ phase in all cases. For double-layered Si_3_N_4_-10 vol % TiN composites (Figure 7), the change of pulsing mode would not vary the relative α-Si_3_N_4_ phase content within both layers. Though a higher degree of phase conversion could be achieved through hot-pressing, due to the slower heating rate, the α-Si_3_N_4_ content difference between the two layers has remained at about 13%, echoing the content gaps seen on SPSed double-layered samples. Therefore, no obvious distinction could be found among the different sintering methods for the double-layered combinations of nano- and micron-TiN with the lower TiN content (10 vol %). An increase in TiN content, from 10 to 30 vol %, would enlarge the α-Si_3_N_4_ content difference between the two layers (Figure 8). Particularly under the continuous current condition, this difference reached a significant level, at 70%. Note that increasing the TiN second phase content in the Si_3_N_4_ matrix has led to a greater retained α-Si_3_N_4_ fraction for most layers, due to the inhibition effect on diffusion within the intergranular liquid phase. However, in the case of the layers with 30 vol % nano- and micron-TiN, and sintered under continuous DC mode, the phase transformation was promoted, dramatically intensifying the differences in the α-Si_3_N_4_ fraction. This phenomenon agreed well with the results of the thermodynamic analysis, and the enhanced performance of the continuous current mode would be reinforced by augmenting the content of the micro-scale conductive second phase. 

Regarding the double-layered combination form of 10-/30- vol % TiN as shown in Figure 9, layers containing more nano-sized TiN still showed a lower α–β transformation degree, independent of the sintering method. Nevertheless, when the TiN particle size increases from the nano-scale to the micron-scale, the same feature exists only in the hot-pressed sample. Under the action of an applied electric field, the relative α-Si_3_N_4_ fractions between adjacent layers were obviously reversed. Together, the results shown in these two double-layered combinations highlight that the favorable effect of the continuous current mode on micron-sized TiN is still in force. The only question remains as to why the enhanced effect of pulsed current mode on nano-TiN is no longer valid on composites with the multi-layered structure.

The α- to β-Si_3_N_4_ conversion occurs by dissolution of α-Si_3_N_4_ into the liquid phase at boundary regions, diffusion of silicon and nitrogen ions within the liquid, and precipitation onto the existing β-Si_3_N_4_ grains. Promoting any of these three stages would enhance the α–β phase conversion process, and hence reduce the conversion activation energy from the thermodynamic perspective. Temperature is always an important factor throughout this process, which would significantly impact the formation and viscosity of the liquid phase, the diffusion coefficient, and the solution saturation. In SPS, the configuration of the graphite mold would also result in quite different heat generation and transfer features, by coupling with the applied electric field, relative to the conventional HP heating process. Consequently, Si_3_N_4_-based ceramics with tailored phase compositions and mechanical properties can be SPS fabricated just by adjusting the graphite punches set-up [5,12,14]. Thus, it could be assumed that the spatial variation in both the layered structures of the samples and the configuration of the graphite mold would change the current intensity and/or propagation path, as well as the electric field distribution. As a consequence, the thermal distribution within the double-layer component is varied, bringing about different observations from those obtained when using the single-layer structure. In this sense, although the multi-layered ceramic components with different phase composition and microstructural features compared with conventionally sintered ones could be prepared using SPS by controlling the current pulsing mode and conductive second phase characteristics, there are requirements for both carefully designed and controlled compositions and geometries for each layer, such as the stacking order, thickness ratio, electrical properties, etc. The validity of these approaches and their implementation needs further investigation.

## 4. Conclusions

In this work, SPS experiments were conducted on the electrical-current-assisted liquid-phase sintering of Si_3_N_4_–TiN composites, controlling both the TiN particle size and current pulsing mode. Their combined effects on the densification behaviors, along with the α-to-β phase conversion of the Si_3_N_4_ matrix, were studied and compared by means of in-situ SPS dilatometric measurements and thermodynamic approach during isothermal sintering. Based on these initial studies, the exploratory application and potential value of using the SPS technique for design and fabrication of double-layered Si_3_N_4_-based ceramics were also demonstrated and discussed.

The reduction of TiN particle size, from 1 µm to 30 nm, contributed to the commencement of sintering shrinkage occurring at a temperature ~200 °C lower. The apparent activation energies for Si_3_N_4_ containing micron- and nano-TiN were 440–650 kJ/mol and 300–560 kJ/mol, respectively. Combining the results demonstrated from the shrinkage rate curves and the apparent *E_a_* evaluation (for both densification and α-to-β phase transformation), it is concluded that the TiN particle size has a significant influence on both the liquid phase particle rearrangement and the solution-diffusion-precipitation processes, by way of the field-induced local heating and possibly electrowetting mechanisms. Regarding the effects of the electric current waveform, the sample with micron-TiN sintered under continuous current conditions showed a lower apparent Ea than that under the pulsed current mode whereas for the specimens containing nano-TiN phase this trend was reversed. It is therefore suggested that the contribution of current mode, on the field-induced densification and phase conversion of the electrically insulating matrix would depend mostly on the particle size and distribution of the electrically conductive phase.

Based on the approach to fabricate double-layered Si_3_N_4_–TiN components, the favorable effect of the continuous current mode on the micron-sized TiN containing Si_3_N_4_ is still in force, but no longer valid for nano-TiN under pulsed current mode. This indicates that the electric-field effect should be dependent not only on the particle size distribution of the conductive phase but also on the geometry of the layer structures. The locally heterogenous electrical properties within multiphase ceramics would control the local electric field (current path) and temperature distributions. This approach offers strong prospects to design multiphase functionally-graded components by taking advantage of particular features of field assisted sintering, although further understanding of the phenomena that occur during the sintering process are needed.
